# Eco-friendly one-pot synthesis of Prussian blue-embedded magnetic hydrogel beads for the removal of cesium from water

**DOI:** 10.1038/s41598-018-29767-y

**Published:** 2018-07-31

**Authors:** Hee-Man Yang, Ju Ri Hwang, Dong Yeop Lee, Kyu Beom Kim, Chan Woo Park, Hee Reyoung Kim, Kune-Woo Lee

**Affiliations:** 1 Decommissioning Technology Research Division, Korea Atomic Energy Research Institute, 989-111 Daedukdaero, Yuseong, Daejeon, 34057 Republic of Korea; 2 Department of Chemical Engineering, Chungnam National University, Daejeon, 34134 Republic of Korea; 3 Department of Chemical Engineering, Hannam University, 70 Hannamro, Daedeok-Gu, Daejeon, 34430 Republic of Korea; 4 Ulsan National Institute of Science and Technology, Ulsan, 689-798 Republic of Korea

## Abstract

A simple one-step approach to fabricating Prussian blue-embedded magnetic hydrogel beads (PB-MHBs) was fabricated for the effective magnetic removal of radioactive cesium (^137^Cs) from water. Through the simple dropwise addition of a mixed aqueous solution of iron salts, commercial PB and polyvinyl alcohol (PVA) to an ammonium hydroxide (NH_4_OH) solution, the formation of hydrogel beads and the encapsulation of PB in beads were achieved in one pot through the gelation of PVA with *in situ*-formed iron oxide nanoparticles as the cross-linker. The obtained PB-MHBs, with 43.77 weight % of PB, were stable without releasing PB for up to 2 weeks and could be effectively separated from aqueous solutions by an external magnetic field, which is convenient for the large-scale treatment of Cs-contaminated water. Detailed Cs adsorption studies revealed that the adsorption isotherms and kinetics could be effectively described by the Langmuir isotherm model and the pseudo-second-order model, respectively. Most importantly, the PB-MHBs exhibited excellent selectivity for ^137^Cs in ^137^Cs-contaminated simulated groundwater (55 Bq/g) with a high removal efficiency (>99.5%), and the effective removal of ^137^Cs from real seawater by these PB-MHBs demonstrated the excellent potential of this material for practical application in the decontamination of ^137^Cs-contaminated seawater.

## Introduction

Due to increased energy demands and concern about global warming, inexpensive and carbon-free nuclear power has been proposed as an alternative to thermal power derived from fossil fuels^1^. However, nuclear power poses many threats to people and the environment, including the leakage of radioactive waste from tanks and the uncontrolled release of enormous amounts of radionuclides caused by nuclear accidents, such as those in Chernobyl and Fukushima^2^. Among the harmful radionuclides generated in nuclear power plants, radioactive cesium (^137^Cs), a strong gamma emitter, is a major component present in radioactive liquid waste and has been involved in nuclear accidents^3^. Moreover, due to its chemical similarity to potassium and high solubility, the ^137^Cs released after a nuclear accident can be easily incorporated into terrestrial and aquatic organisms^4,5^. Unfortunately, the current methods for treating radioactive liquid waste in the nuclear industry, including solvent extraction, chemical precipitation, membrane processing, coagulation, electrodialysis and ion exchange, are not suitable for the remediation of ^137^Cs-contaminated aqueous media after a nuclear accident due to the large volume of radioactive waste needed to be processed and the extremely low concentration of ^137^Cs compared to that of competitive ions, such as Na^+^, K^+^, Ca^2+^, and Mg^2+^ ^6^. Thus, highly selective Cs adsorption is the most suitable method for remediating ^137^Cs-contaminated water due to its simplicity and efficiency in the treatment of large volumes of radioactive liquid waste^7^. A variety of Cs^+^ adsorbents, such as zeolites^8^ and silicotitanates^9^, have been previously investigated in the removal of ^137^Cs from water. However, these adsorbents are either expensive or lack the efficiency required for large-scale remediation due to their low Cs selectivity.

Prussian blue (PB, ferric hexacyanoferrate), one of the metal hexacyanoferrates, with high selectivity for Cs, was effectively used to remove ^137^Cs after the Chernobyl nuclear accident and the Goiania accident^10,11^ and has been approved by the U.S. Food and Drug Administration for the treatment of radioactive Cs poisoning in humans, meaning that its use is likely safe for both humans and the environment^12^. However, the PB prepared by precipitation is usually an ultra-fine powder of submicron size, which limits its direct use in the large-scale remediation of contaminated environments due to the difficulty of separating the PB using centrifugation or filtration after ^137^Cs sorption^13^.

Various efforts have been made to efficiently use PB in large-scale applications. The several strategies reported to overcome this drawback can be categorized into two groups. One involves the immobilization of PB on various support materials. For example, the surfaces of mesoporous silica^14,15^ and carbon materials^16,17^ were functionalized with PB, and various polymer matrices, such as chitin^18,19^, alginate^20,21^, polyacrylonitrile^22^, and polyacrylic acid^23^, were used to encapsulate PB. The other approach is to use magnetic particles to magnetically separate the adsorbent after ^137^Cs sorption. In such methods, various metal hexacyanoferrates, including PB, were coated on the surface of magnetic nanoparticles (MNPs) using functional coating materials^24–26^. However, these two approaches usually require multistep processes with complicated synthetic procedures, which limits their practical application due to the difficulty of mass production.

In this study, a simple and convenient method for synthesizing PB-embedded magnetic hydrogel beads (PB-MHBs) for the removal of ^137^Cs from water, including seawater, was investigated. The PB-MHBs were simply fabricated by the facile dropwise addition of a mixed aqueous solution of iron salts, commercial PB and polyvinyl alcohol (PVA) to an ammonium hydroxide (NH_4_OH) solution. During the formation of the PVA-iron oxide nanoparticle-based hydrogel bead, the commercial PB was encapsulated in the hydrogel network, resulting in the facile generation of MHBs containing PB as a Cs adsorbent. Thus, these PB-MHBs prepared using our one-pot method are suitable for practical applications because the synthetic strategy is easily scalable under ambient conditions and uses only eco-friendly and low-cost precursors.

## Results

### Synthesis and characterization of PB-MHBs

**Figure 1:**
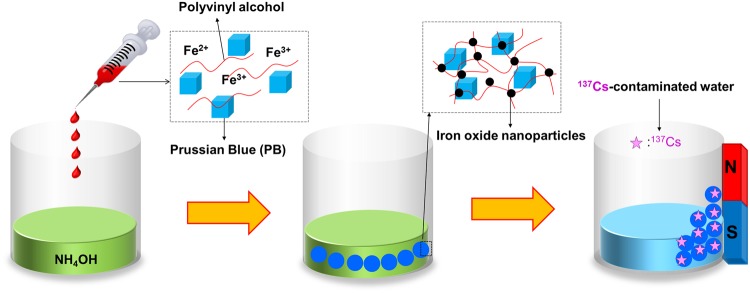
Synthetic procedure used to prepare the PB-MHBs and their use in the removal of radioactive Cs.

As shown in Fig. 1, the iron oxide nanoparticles made the PB-embedded hydrogel magnetically responsive. The preparation of superparamagnetic iron oxide nanoparticles by a chemical co-precipitation method involving ferric and ferrous ions in an alkali solution is well known^27^. The detailed co-precipitation reaction is as follows.

1 $$2{{\rm{Fe}}}^{3+}+{{\rm{Fe}}}^{2+}+8{{\rm{OH}}}^{-}\to {{\rm{Fe}}}_{3}{{\rm{O}}}_{4}+4{{\rm{H}}}_{2}{\rm{O}}$$

**Figure 2:**
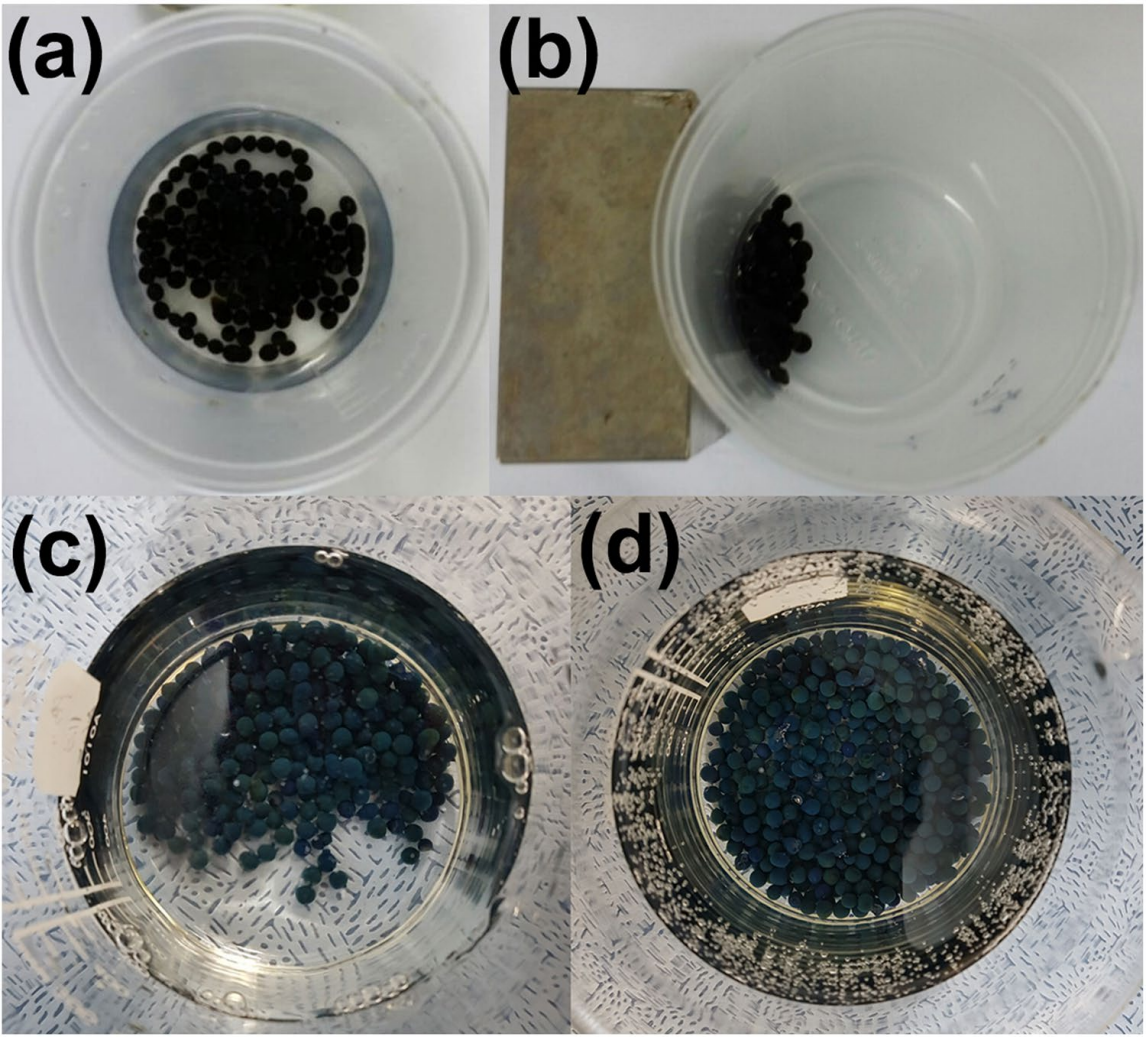
(**a**) Photographs of Prussian blue-embedded magnetic hydrogel beads (PB-MHBs) after reaction with an NH_4_OH solution (pH 11.3); (**b**) the separated solution after placement of a magnet next to the original dispersion of PB-MHBs; and photographs of (**c**) PB-MHBs-2 and (**d**) PB-MHBs-3.

**Figure 3:**
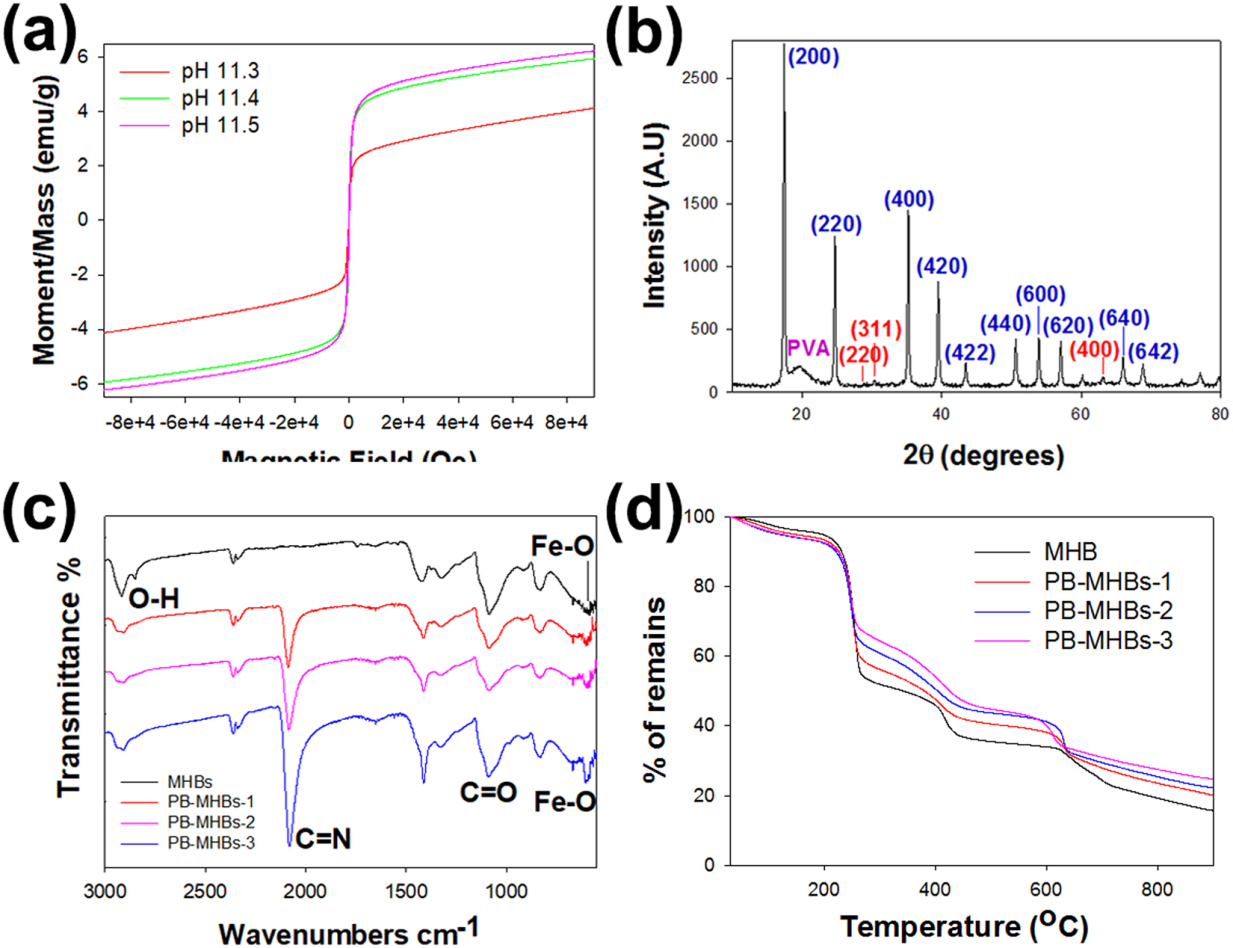
Magnetization curves obtained from (**a**) PB-MHBs fabricated from NH_4_OH solutions with different pH values; (**b**) X-ray diffraction patterns obtained from PB-MHBs-3; (**c**) FTIR spectra of MHBs without PB (MHBs) and PB-MHBs-1, -2, and -3; and (**d**) TGA curves obtained from MHBs without PB (MHBs) and PB-MHBs-1, -2, and -3.

However, the iron oxide nanoparticles formed by co-precipitation easily aggregate. To prevent the aggregation of the iron oxide nanoparticles in the hydrogel beads, PVA was selected as the polymeric component of the hydrogel because it can adsorb iron ions through its hydroxyl groups to form a complex homogeneous structure in a mixed solution and subsequently acts as a stabilizer of the iron oxide nanoparticles^28^. Although the complete precipitation of Fe_3_O_4_ is known to occur at pH values between 8 and 14^29^, the pH conditions for the fabrication of the PVA-iron oxide nanoparticles-based hydrogel needed to be investigated. Therefore, a mixed solution of PVA, iron salts and commercial PB (50 mg) was added dropwise to solutions of NH_4_OH with various pH values. Figure S1 shows that when the pH was below 11.3, the beads had an irregular shape, and the solution became yellow, indicating that some iron ions were released from the beads. However, the PB-MHBs fabricated at pH values higher than 11.3 had spherical shapes, and the solution was transparent without turning yellow, indicating the successful formation of iron oxide nanoparticles without the release of iron ions (Figs S1 and 2a). Therefore, the minimum pH for the fabrication of PB-embedded hydrogel beads was found to be 11.3. Additionally, the calculated average size of PB-MHBs was 33.8 mm. The X-ray powder diffraction (XRD) pattern obtained from the PB-MHBs fabricated at pH values of 11.3, 11.4, and 11.5 exhibited the characteristic peaks of both PB and Fe_3_O_4_, indicating the successful formation of magnetic particles and encapsulation of PB in the hydrogel beads (Fig. S2). Moreover, Fig. 2b shows that the PB-MHBs fabricated at pH 11.3 could be easily collected from water by an external magnet. The magnetic properties of the various PB-MHBs were further analyzed using a vibrating sample magnetometer (VSM). Figure 3a shows that all the beads had superparamagnetic behavior due to the presence of Fe_3_O_4_ nanoparticles in the beads. The saturation magnetizations (*M*_*s*_ values) of the PB-MHBs fabricated at pH values of 11.3, 11.4, and 11.5 were 4.13 emu g^–1^, 5.94 emu g^–1^ and 6.23 emu g^–1^, respectively. The differences between the three types of PB-MHBs can be attributed to differences in the amount of Fe_3_O_4_ nanoparticles in the hydrogel beads because higher pH values generally produce larger amounts of nanoparticles, resulting in higher *M*_*s*_ values. Based on the successful one-pot fabrication results, the PB-MHBs could be fabricated in a large quantity, which is crucial for their practical applications.

### Effect of the PB amount in the hydrogel beads

The content of PB used as a Cs adsorbent in the hydrogel beads is a key parameter for the practical application of the beads in the removal of ^137^Cs from contaminated environments. Thus, a series of PB-MHBs were prepared by adjusting the feed weight ratio of PB to PVA from 10% to 60% with a fixed amount of PVA (500 mg, Table 1). Note that when more than 300 mg of PB was added, the fabricated PB-MHBs became very brittle and had poor mechanical properties (data not shown). This result was caused by a too-high PB content leading to the formation of PB-MHBs with a low degree of cross-linking, resulting in low mechanical stability. Compared with the PB-MHBs fabricated with a feed ratio of 10% (Fig. 2a), the color of the PB-MHBs fabricated with feed ratios of 40% (PB-MHBs-2) and 60% (PB-MHBs-3) were a darker blue due to the increased content of the blue-colored PB in the beads (Fig. 2c and d). Moreover, the intensity of the characteristic peaks of PB in the XRD pattern of PB-MHBs-3 dramatically increased due to their increased content of PB (Fig. 3b) compared with the XRD pattern of the PB-MHBs fabricated with a feed ratio of 10% (Fig. S2). FTIR analysis revealed the successful formation of PB-MHBs (Fig. 3c) because strong absorption peaks corresponding to the stretching vibration of a cyanide group (-C≡N-) of PB were observed at 2065 cm^−1^ in the FTIR spectra of PB-MHBs-1, -2, and -3.

**Table 1:** Weight % of each component of the PB-MHBs calculated from the results of the TGA analysis.

	*Feed ratio (PB: PV)*	*Remaining % from TGA*	*Fe*_3_ *O*_4_	*Commercial PB* *(KFeFe(CN)*_6_ *·x H*_2_ *O)*		*PVA*
				*Inorganic comp*.	*Organic comp*.	
MHBs	0	15.65 wt%	15.65 wt%	—	—	84.35 wt%
PB-MHBs-1	20%	20.10 wt%	12.29 wt%	7.81 wt%	13.64 wt%	66.26 wt%
				21.45 wt%		
PB-MHBs-2	40%	22.22 wt%	10.69 wt%	11.53 wt%	20.14 wt%	57.64 wt%
				31.67 wt%		
PB-MHBs-3	60%	24.73 wt%	8.80 wt%	15.93 wt%	27.84 wt%	
				47.43 wt%		

**Figure 4:**
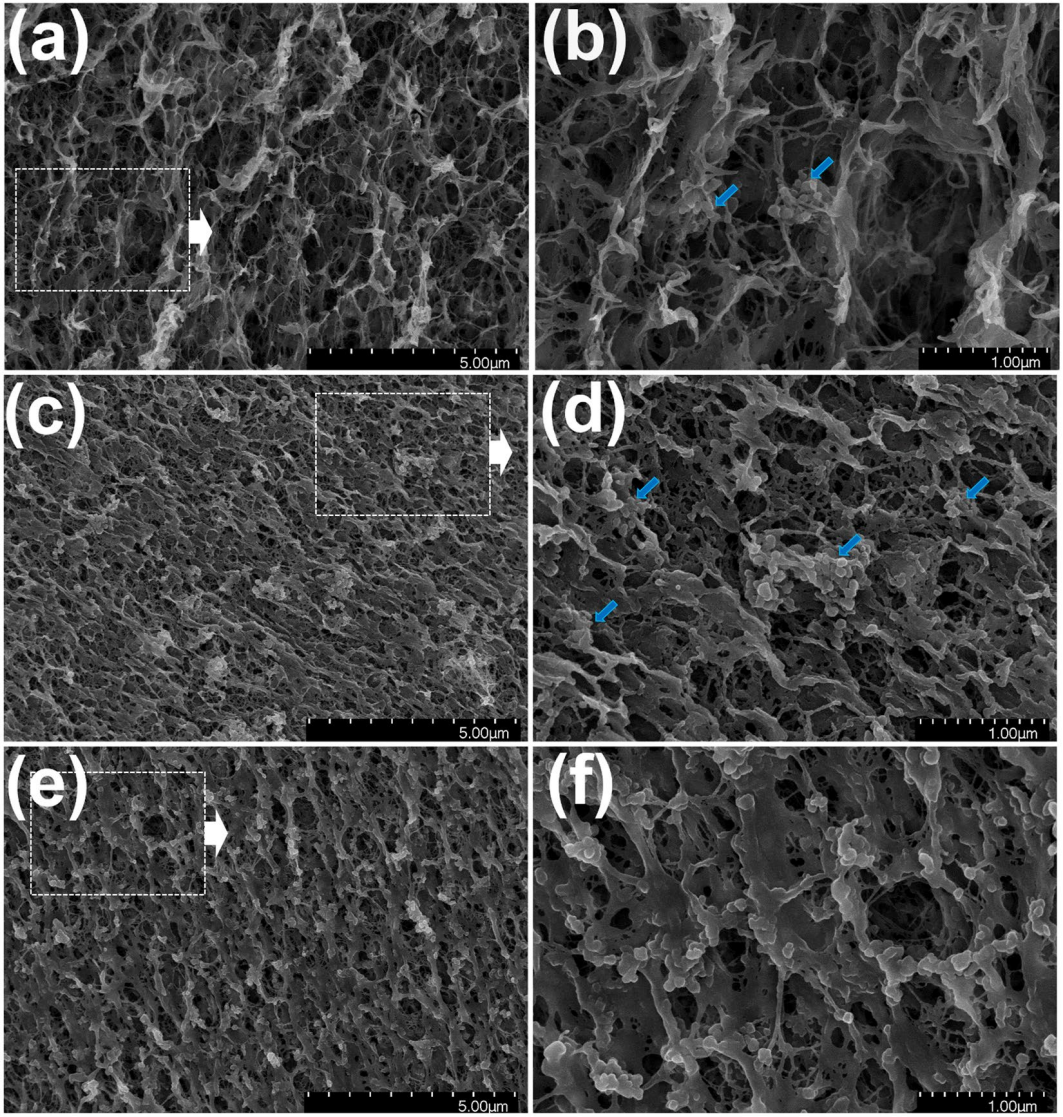
Scanning electron microscopy (SEM) images of PB-MHBs-1 (**a,b**), PB-MHBs-2 (**c,d**), and PB-MHBs-3 (**e,f**).

To gain more insight into the structure of the PB-MHBs, PB-MHBs-1, -2, and -3 were characterized by scanning electron microscopy (SEM) measurements. Figure S3 shows an SEM image of the commercial PB and MHBs fabricated without PB. The commercial PB particles had an irregular ellipsoidal shape with a wide size distribution from 50 nm to 100 nm (Fig. S3a). Figure S3b shows that the MHBs had a microporous 3D network, which is correlated to the Cs adsorption capacity of PB-MHBs because it is beneficial for transporting Cs ions across the bead. Additionally, Fe atoms in EDX data and mapping of MHBs without PB revealed the successful gelation of PVA with *in situ*-formed iron oxide nanoparticles (Figure S3c and d). Figure 4 shows SEM images of PB-MHBs-1, -2, and -3 at different magnifications. As shown in Fig. 4, the open microporous structure of cross-linked PBA-Fe_3_O_4_ was maintained even after the addition of PB, and the PB particles in all the PB-MHBs were successfully adhered onto the PVA-Fe_3_O_4_ network. As shown in Fig. 4f, the PB in the PB-MHBs was uniformly dispersed with dense packing. However, when the feed weight ratio of PB to PVA was higher than 40%, a less porous and more rigid wall structure was formed. This might be attributed to the high content of PB in the PB-MHBs.

To further investigate the contents of PB, Fe_3_O_4_ nanoparticles and PVA in PB-MHBs-1, -2, and -3, TGA measurements were performed (Fig. 3d). For the MHBs, the residual material consisted entirely of Fe_3_O_4_ because the PVA in the beads was completely decomposed upon heating to 900 °C, whereas the residual material in the case of the PB-MHBs consisted of both Fe_3_O_4_ and the inorganic component of PB because only the organic component of PB could decompose. According to the TGA data for the MHBs, the wt% of Fe_3_O_4_ and PVA were 15.65 wt% and 84.35 wt%, respectively, corresponding to a weight ratio of PVA to Fe_3_O_4_ of 5.39. Assuming that the weight ratio of PVA to Fe_3_O_4_ was maintained when PB was encapsulated in the beads, the inorganic and organic components of PB in each type of PB-MHB could be calculated from the weight ratio of PVA to Fe_3_O_4_ (5.39), the chemical composition of PB (KFe_2_(CN)_6_), and the remaining wt% of each type of PB-MHB, which corresponded to the combined wt% of Fe_3_O_4_ and the inorganic component of PB. The calculated wt% of each component of the PB-MHBs is listed in Table 1. As the feed ratio of PVA to PB was increased, the wt% of PB increased, whereas the wt% of Fe_3_O_4_ decreased. In addition, the maximum wt% of PB was approximately 43.77% in the case of PB-MHBs-3, which is similar to the wt% of PVA, demonstrating the excellent encapsulating ability of the PB-MHBs.

PB-MHBs-3, which had the highest amount of PB, exhibited obvious magnetic behavior with some coercive force (Fig. S4), which is consistent with previously reported magnetic adsorbents with a high content of PB^30^. The *M*_*s*_ value of PB-MHBs-3 was 2.17 emu/g, which was lower than that obtained for the PB-MHBs fabricated with a feed ratio of 10% (4.13 emu/g). This lower value is attributed to the higher content of PB in PB-MHBs-3 relative to the others because PB has no magnetization, consistent with the TGA results. Although PB-MHBs-3 had the lowest *M*_*s*_ value because they had the lowest content of Fe_3_O_4_, we anticipated that the adsorption ability of PB-MHBs-3 would be better than that of the others because PB-MHBs-3 had the highest content of PB. Based on the above TGA and VSM results, the magnetization of PB-MHBs can be easily controlled by adjusting the content of PB according to practical demands to achieve excellent Cs adsorption ability.

### Stability of PB-MHBs-3

In practical applications, the leaching of adsorbent components into water is undesirable. PB is known to have a strong absorption peak at 690 nm. As shown in Figure S5, the absorption band of PB was absent in the UV-vis spectrum of PB-MHBs-3 for 2 weeks, which confirmed that PB was not released from the PB-MHBs. This may be attributed to the wrapping effect of PB with PVA cross-linked with iron oxide as a binding matrix^20^.

The release of Fe_3_O_4_, the cross-linker for PVA gelation in PB-MHBs, was evaluated by measuring the Fe after suspending PB-MHBs-3 in water for various times (Table S3). Up to 4 days, the Fe leaching was below 0.25%, and it was maintained below 0.3% for up to 2 weeks. Overall, the PB-MHBs were stable even after 2 weeks of suspension without the release of either PB or iron oxide from PB-MHBs. Therefore, the PB-MHBs should be sufficiently stable for practical applications.

### Cs removal performance of the PB-MHBs

The Cs removal performance of the PB-MHB sample, in terms of the removal efficiency and distribution coefficient (K_d_), was first evaluated using inactive Cs according to the following equation:

2 $${\rm{R}}=(({{\rm{C}}}_{0}\mbox{--}{{\rm{C}}}_{{\rm{e}}}))/{{\rm{C}}}_{0}\times 100,$$

3 $${{\rm{K}}}_{{\rm{d}}}=(({{\rm{C}}}_{{\rm{0}}}\mbox{--}{{\rm{C}}}_{{\rm{e}}}))/{{\rm{C}}}_{{\rm{e}}}\times {\rm{V}}/{\rm{m}},$$

where C_o_ and C_e_ are the initial and equilibrium concentrations of Cs in the solution, V is the volume of the solution (10 mL), and m is the mass of the adsorbent (10 mg). As shown in Table S1, the Cs removal efficiencies of MHBs, PB-MHBs-2, and PB-MHBs-3 were found to be 0.80%, 27.76%, and 32.88%, respectively. Additionally, the K_d_ values of PB-MHBs-2 (384.02 mL/g) and PB-MHBs-3 (489.687 mL/g) were 45.99 and 58.64 times higher than that of MHBs (8.346 mL/g). These results indicated that Cs sorption by the PB-MHBs was entirely attributed to PB having strong binding affinity toward Cs, even though the MHBs and all the PB-MHBs had the same microporous 3D network structure. The adsorption ability of PB for Cs is well known to be caused by the regular lattice spaces surrounded by the cyanide-bridged metal and the proton-exchange mechanism of the specific Cs adsorption^31^. Figure S6a shows an SEM image of PB-MHBs-3 after the adsorption reaction with Cs. The porosity of the PB-MHBs was maintained, indicating that the adsorption of Cs did not change the structure of the PB-MHBs. EDX data revealed the successful adsorption of Cs onto PB-MHBs. Furthermore, Cs mapping showed the homogeneous dispersion of Cs onto PB-MHBs-3, indicating that Cs was completely diffused onto the PB-MHBs with a porous network structure, which is a prerequisite for a good adsorbent material.

**Figure 5:**
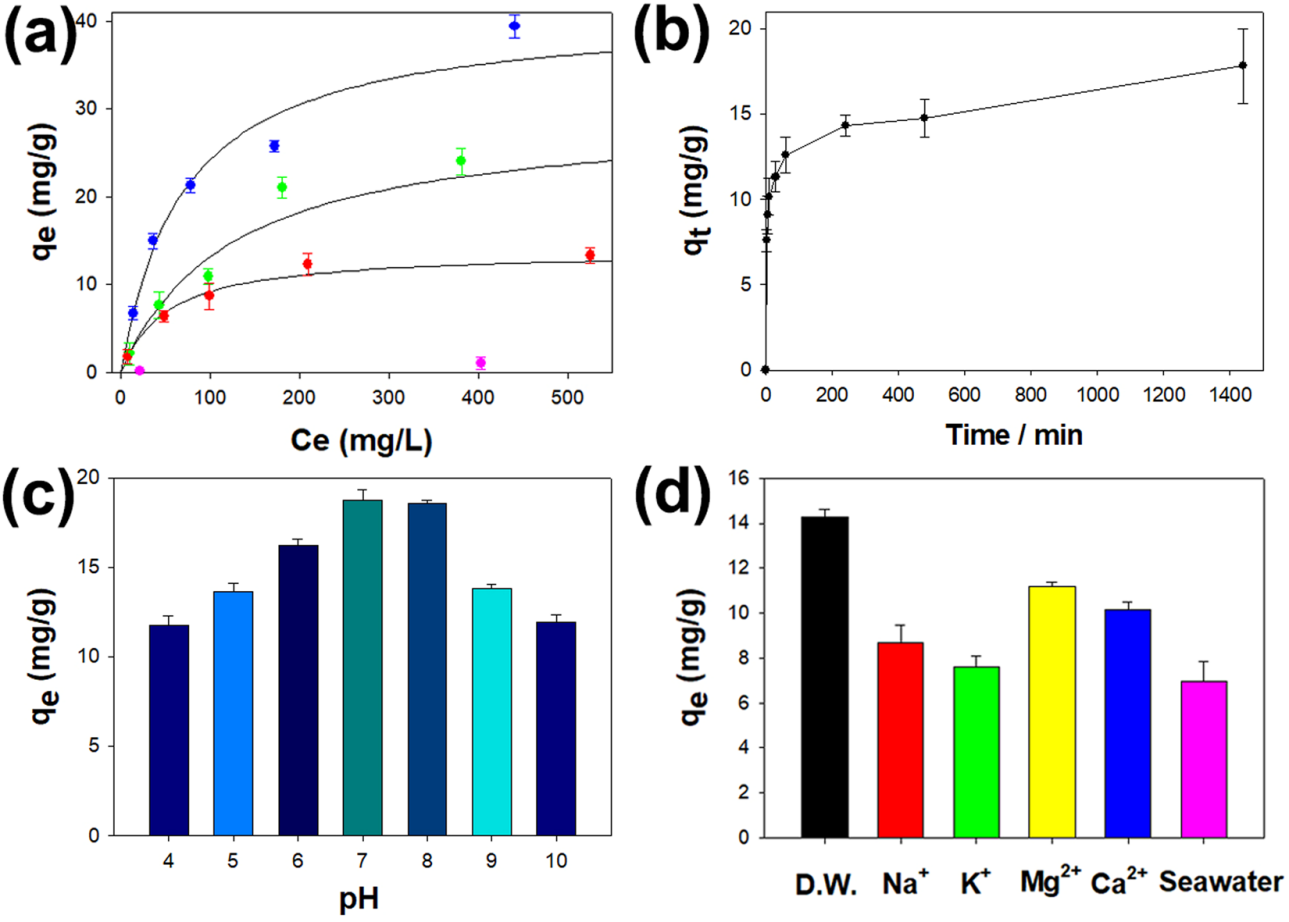
(**a**) Adsorption isotherm data obtained from the various PB-MHBs (red: PB-MHBs-1, green: PB-MHBs-2, blue: PB-MHBs-3, pink: MHBs; the curve fits were obtained from the Langmuir adsorption isotherm model). Influence of (**b**) contact time, (**c**) pH, and (**d**) competing ions on the Cs adsorption capacity of PB-MHBs-3.

The adsorption capacities of PB-MHBs-1, -2 and -3 were evaluated by measuring the adsorption isotherms. Figure 5a shows the equilibrium amounts of inactive Cs adsorbed onto PB-MHBs-1, -2 and -3 from solutions with different initial concentrations versus the concentration of Cs after equilibration. The experimental isotherm data were analyzed as Langmuir isotherms^32^. The expression for the Langmuir isotherm is given by the following equation:

4 $${\rm{1}}/{{\rm{q}}}_{{\rm{e}}}=1/{{\rm{q}}}_{{\rm{\max }}}+1/({{\rm{q}}}_{{\rm{\max }}}{{\rm{bC}}}_{{\rm{e}}}),$$

where q_e_ and q_max_ are the equilibrium adsorption capacity and the maximum adsorption capacity, respectively. Here, b is the Langmuir constant related to the energy of adsorption. The values of q_max_ and b could be calculated from the intercept and the slope, respectively, of a linear plot of 1/q_e_ versus 1/C_e_. The isotherm data were fit well by the Langmuir model according to the high R^2^ values (Fig. 5a and Table S1), suggesting the monolayer adsorption of Cs onto the PB-MHB surface. The q_max_ values of PB-MHBs-1, -2, and -3 were 13.87 mg/g, 29.49 mg/g, and 41.14 mg/g, respectively. PB-MHBs-3 had the highest q_*max*_ value due to having the highest content of PB. To compare the Cs removal results of the PB-MHBs to those from other reports, the q_max_ values of various previously reported metal ferrocyanide-based hydrogel beads were determined, as summarized in Table 2. PB-MHBs-3 provided a 2.7-fold higher q_max_ value than cobalt hexacyanoferrate-modified gel beads^20^ due to the high content of commercial PB in PB-MHBs-3. Although the q_max_ value of PB-MHBs-3 was similar to that of PB encapsulated in chitin beads^18^ and lower than that of PB and carbon nanotubes encapsulated in alginate beads^21^, our PB-MHBs are magnetically responsive, facilitating their magnetic separation after application, whereas the others cannot respond to an external magnet. In particular, the q_max_ value of PB-MHBs-3 fabricated using commercial PB without any other functional materials, such as grapheme oxide (GO), provide a similar q_max_ value to that of magnetically responsive PB/Fe_3_O_4_-coated GO encapsulated in alginate beads, which used GO as a supporting material to immobilize the PB and Fe_3_O_4_ nanoparticles^33^.

**Table 2:** Comparison of the maximum Cs adsorption capacities of various PB-based hydrogel beads.

Sorbent	q_max_ (mg/g)	Separation	Ref.
Cobalt ferrocyanide-modified gel beads	15.0	—	Dwivedi *et al*.^20^
PB encapsulated in chitin beads	42.45	—	Vincent *et al*.^18^
PB encapsulated in alginate beads	131.57	—	Vipin *et al*.^21^
PB- and MNP-coated GO encapsulated in alginate beads	43.516	Magnetic	Yang *et al*.^33^
PB-MHBs-3	41.15	Magnetic	This work

Figure 5b plots the amount of Cs adsorbed onto PB-MHBs-3 as a function of contact time. Over 24 h was required for PB-MHBs-3 to reach adsorption equilibrium. The slower adsorption of PB-MHBs-3 may be attributed to the use of commercial PB, which is known to have slow kinetics due to limitations in diffusion^34^. However, 80% of the total amount of adsorbed Cs was adsorbed within a few hours of contact, as shown in Fig. 5b, indicating that PB-MHBs-3 had a high affinity for Cs. To investigate the sorption process, the kinetic data were analyzed using the pseudo-second-order model. The related equations are given by equation (5) as follows:

5 $${\rm{t}}/{{\rm{q}}}_{{\rm{t}}}=1/{{\rm{q}}}_{{\rm{e}}}{\rm{t}}+1/({{\rm{k}}}_{{\rm{2}}}\,{{{\rm{q}}}_{{\rm{e}}}}^{2}),$$

where q_t_ and q_e_ are the adsorption capacities at equilibrium and at time t (mg/g), respectively, and k_2_ is the rate constant of the pseudo-second-order model (g/mg·min). When the experimental data were fit by linear regression (Figure S4 and Table 3), the data were fit well by the pseudo-second-order kinetic model according to the high R^2^ values, and the rate-limiting step was chemical adsorption. This result is consistent with the results reported for other PB-embedded hydrogels beads^20,21^.

**Table 3:** Kinetic parameters fitted by the pseudo-second-order model.

*k*_2_ *(g mg*^*−1*^ *min*^*−1*^)	*q*_*e*_ *(mg/g)*	*R* ^2^
0.002446	17.73	0.9949

### Effects of pH and competing ions on Cs adsorption by PB-MHBs-3

The effect of pH on the adsorption performance of PB-MHBs-3 was next explored. The experiments were conducted by mixing 10 mg of PB-MHBs-3 with 10 mL of a Cs solution (100 ppm) at various pH values. HCl and NaOH were used to adjust the pH. Figure 5c plots the amount of Cs adsorbed per unit weight of PB-MHBs-3 (q_e_, mg/g) at various pH values. PB-MHBs-3 showed the maximum value at pH 7, and a decrease in the adsorption capacity was observed in acidic and alkaline conditions. This decrease at acidic pH was related to the competition that occurs between H^+^ and Cs^+^ due to the protonation of the ion exchange sites in the commercial PB of the PB-MHBs^16,35^. At an alkaline pH range from 9 to 10, the q_e_ value also decreased because some fraction of the commercial PB in PB-MHBs-3 decomposed under alkaline conditions^16,30^.

The effects of competing ions on the adsorption performance of PB-MHBs-3 (10 mg/10 mL) were also explored. Ions that are abundant in seawater, such as Na^+^ and K^+^, and ions that coexist with Cs^+^ in nuclear liquid waste, such as Ca^2+^ and Mg^2+^, were chosen as competing ions. Figure 5d shows the change in q_e_ after treatment of a Cs solution with an initial concentration of 100 ppm with PB-MHBs-3 in the presence of other competing ions at a higher concentration of 3000 ppm. Compared with the q_e_ value obtained for the aqueous medium without any competing ions, the q_e_ values decreased in the presence of other competing ions, which is consistent with the results of previous reports^24^. The q_e_ value was the lowest in the presence of K^+^ (8.68 mg/g), which is related to the hydration radii of the competing ions because the hydration radius of K^+^ (3.3 Å) is closer to that of Cs^+^ (3.25 Å) than are the radii of Na^+^ (3.6 Å), Ca^2+^ (4.1 Å), and Mg^2+^ (4.25 Å), resulting in the highest competition between Cs^+^ and K^+^ in binding to PB^25^. For practical applications, the Cs removal performance of PB-MHBs-3 was further examined in simulated seawater (11,000 ppm Na^+^, 1300 ppm Mg^2+^, and 500 ppm Ca^2+^ and K^+^) (Fig. 5d). Although the adsorption capacity was reduced in seawater, the effective q_e_ value (6.94 mg/g) in the presence of an approximately 125-fold higher concentration of competing cations than Cs demonstrated that PB-MHBs-3 has potential utility in the remediation of Cs-contaminated seawater.

### Radioactive ^137^Cs decontamination using PB-MHBs-3

We finally sought to determine the ability of PB-MHBs-3 to remove very low concentrations of ^137^Cs from various aqueous media because most radio-contaminated liquid wastes generated during the operation of nuclear power plants and the contaminated groundwater or seawater produced after nuclear accidents have low levels of ^137^Cs. For the radioactive tests, various ^137^Cs-contaminated aqueous media, including simulated groundwater and real seawater, were prepared with a radioactivity of 54.79 Bq/g (approximately 0.0171 ppb). The ^137^Cs removal performance of PB-MHBs-3 was evaluated in terms of the decontamination factor (DF) and removal efficiency (R), as defined by the following equations:

6 $${\rm{R}}=({{\rm{A}}}_{{\rm{0}}}-{{\rm{A}}}_{{\rm{f}}})/{{\rm{A}}}_{{\rm{f}}}\times 100 \% ,$$

7 $${\rm{DF}}={{\rm{A}}}_{0}/{{\rm{A}}}_{{\rm{f}}},$$

where A_0_ and A_f_ are the ^137^Cs activities in the initial and final solutions, respectively, after treatment with PB-MHBs-3. The DF and R values are listed in Table 4. The ^137^Cs activity in distilled water was significantly decreased after treatment with PB-MHBs-3, indicating that PB-MHBs-3 successfully removed the extremely small amount of ^137^Cs (0.0171 ppb) from the water. PB-MHBs-3 provided an excellent ^137^Cs removal efficiency of 98.375% even at low adsorbent concentrations (0.1 mg/mL). Notably, the final activity of the ^137^Cs solution was further decreased as the concentration of PB-MHBs-3 was increased, yielding an excellent removal efficiency that exceeded 99.849% and a DF that exceeded 660.

**Table 4:** Removal of ^137^Cs from water by PB-MHBs-3 (A_o_: initial activity; A_f_: final activity after treatment).

Adsorbent conc.	Medium	A_o_ (Bq/g)	A_f_ (Bq/g)	R	DF
0.1 mg/mL	Distilled water	54.7936	0.8903	98.375%	61.545
1 mg/mL	Distilled water	54.7936	0.0829	99.849%	660.917
0.1 mg/mL	Simulated groundwater	54.7936	1.2301	97.755%	44.541
1 mg/mL	Simulated groundwater	54.7936	0.2663	99.514%	205.74
1 mg/mL	Real seawater	54.7936	1.7842	96.744%	30.7085

The ^137^Cs removal performance of PB-MHBs-3 was further examined in simulated groundwater (125 ppm Na^+^, 25 ppm Ca^2+^, 10 ppm Mg^2+^, and 5 ppm K^+^) (Table 4). The R value in simulated groundwater was slightly decreased relative to that in distilled water, from 98.375% to 97.755% for 0.1 mg/mL PB-MHBs-3 and from 99.849% to 99.514% for 1 mg/mL PB-MHBs-3, due to the competing effects of Na^+^, Ca^2+^, Mg^2+^, and K^+^. However, the competing effects were negligible, even when the concentration of competing ions (total 165 ppm) was significantly higher than the initial concentration of ^137^Cs (0.0171 ppb), which is contrary to the results from the competing ion effects with non-radioactive Cs (Fig. 5d). This difference can likely be attributed to the difference in the initial concentration of Cs between the non-radioactive Cs experiments (100 ppm) and the ^137^Cs experiments (0.0171 ppb). Based on these results, PB-MHBs-3 exhibited excellent selective adsorption of ^137^Cs because the conditions of the radioactive test more closely mimicked those of the real environment after a nuclear accident; the concentration of Cs diffused from nuclear fallout is typically significantly lower than the concentrations of cations in any environment.

Encouraged by the results from the radioactive experiments, ^137^Cs decontamination of seawater was examined using real seawater collected from the Korean East Sea with the same initial activity as that in the above performance evaluation. The DF and R values are listed in Table 4. As expected, the R value decreased relative to those in distilled water and simulated groundwater due to the coexistence of many competing ions in real seawater. However, an excellent R value exceeding 96.7% and a DF exceeding 30 were maintained, indicating the effective removal of ^137^Cs from real seawater. Therefore, the PB-MHBs are a good candidate for ^137^Cs removal from various ^137^Cs-contaminated waters, including groundwater and seawater.

## Discussion

In summary, we successfully demonstrated a simple and scalable synthesis of PB-MHBs for the effective removal of radioactive Cs from water with the ability to separate the beads magnetically. The gelation of PVA with *in situ*-formed Fe_3_O_4_ nanoparticles as a cross-linker was applied to encapsulate commercial PB in hydrogel beads. The minimum pH for the fabrication of PB-MHBs was 11.3. All the PB-MHBs prepared with various feed weight ratios of PB to PVA showed a magnetic response suitable for magnetic separation of the beads from water. The adsorption behavior of these PB-MHBs was fit well by both the Langmuir isotherm model, with a maximum Cs adsorption capacity of 41.15 mg/g, and by the pseudo-second-order kinetic model. In the radioactive tests, the PB-MHBs exhibited excellent selectivity for ^137^Cs with a removal efficiency exceeding 99.5%, even in simulated groundwater, and effectively removed ^137^Cs from real seawater. Therefore, the PB-MHBs demonstrated excellent potential for the treatment of ^137^Cs-contaminated water, and this synthetic approach for PB-MHBs can be extended to prepare MHBs for the removal of other radionuclides by simply adding functional adsorbents to the PVA solution.

## Methods

### Chemicals

Polyvinyl alcohol (MW 146,000~186,000), iron(II) chloride tetrahydrate (FeCl_2_·4H_2_O), iron(III) chloride (FeCl_3_), ammonium hydroxide (NH_4_OH, 25 wt%), and Prussian blue (PB, KFeFe(CN)_6_, soluble form) were purchased from Sigma-Aldrich and were used as received.

### Fabrication of PB-MHBs

First, 500 mg of PVA and a certain amount of PB (50 mg, 100 mg, 200 mg, or 300 mg) were dissolved in water by stirring and heating at 80 °C. Then, 68.14 mg of FeCl_2_·4H_2_O and 111.86 mg of FeCl_3_ were added to the solution. After stirring for 30 min, the mixture was gradually added dropwise to an NH_4_OH solution with a pH value of 11.3 using a pipette with a 0.2-mL-maximum-volume pipette tip. The immediately formed hydrogel beads were repeatedly washed with deionized water.

### Characterization

FTIR spectra were recorded using a Spectrum GX & Auto Image instrument (Perkin-Elmer). XRD patterns were collected on a high-resolution X-ray diffractometer (model: Smartlab, Rigaku). TGA was performed using a Setsys 16/18 instrument (Setaram, France). The saturation of magnetization was evaluated using a VSM (Lakeshore, model 955287(A)). SEM images were obtained using a low-voltage field emission SEM instrument (model: Merlin compact, Zeiss).

### Cs removal performance test using inactive Cs

The adsorption kinetics and isotherms were investigated in a batch experiment at 25 °C. The adsorption isotherm study was conducted by mixing 10 mg of various PB-MHBs with 10 mL of an aqueous solution containing various concentrations of inactive Cs (10–500 ppm). After the mixture was shaken for 24 h, the PB-MHBs were separated using a magnet, and the initial and residual Cs concentrations were analyzed using inductively coupled plasma mass spectrometry (ICP-MS). The initial concentration of the inactive Cs solution used for the kinetic study was 100 ppm. A total of 10 mg of the PB-MHBs was mixed with 10 mL of the abovementioned Cs-containing solution for different times (5–1400 min). The residual Cs concentration was analyzed using ICP-MS.

### Radioactive studies

The radioactive experiments were conducted by mixing the PB-MHBs with an aqueous solution of ^137^Cs without or with 125 ppm of Na^+^, 25 ppm of Ca^2+^, 10 ppm Mg^2+^, and 5 ppm of K^+^ to simulate groundwater. After stirring for 24 h, PB-MHBs-3 were collected. The radioactivity of ^137^Cs before and after treatment with PB-MHBs-3 was measured using an HPGe detector (Canberra, USA).

## Electronic supplementary material

supplementary information
