# Supplementary material for: Eco-friendly one-pot synthesis of Prussian blue-embedded magnetic hydrogel beads for the removal of cesium from water
Source: Sci Rep. 2018 Jul 31;8:11476. doi: 10.1038/s41598-018-29767-y (PMC6068103; doi:10.1038/s41598-018-29767-y)
Supplement: Supplementary file 1 — supplementary information [file 41598_2018_29767_MOESM1_ESM.doc]

**Eco-friendly one-pot synthesis of Prussian blue-embedded magnetic hydrogel beads for the removal of cesium from water**

Hee-Man Yang ^a^,* Ju Ri Hwang,^a,b^ Dong-Yeop Lee^a,c^, Kyu Beom Kim^a,c^ Chan Woo Park^a^, Hee Reyoung Kim^d^, Kune-Woo Lee^a^,

Table S1. Cs removal performance of PB-MHBs and MHBs (without PB).

|  | *C_o_* | *C_f_* | *Removal efficiency* | *K_d_* |
| --- | --- | --- | --- | --- |
| MHBs | 21.26 ppm | 21.09 ppm | 0.80 % | 8.35 |
| PB-MHBs-2 | 20.53 ppm | 14.83 ppm | 27.76 % | 384.02 |
| PB-MHBs-3 | 20.53 ppm | 13.78 ppm | 32.88 % | 489.69 |

**Table S2. Langmuir adsorption isotherm model fit parameters.**

|  | *B* | *q_max_ (mg/g)* | *R^2^* |
| --- | --- | --- | --- |
| PB-MHBs-1 | 0.019524 | 13.87 | 0.9989 |
| PB-MHBs-2 | 0.008072 | 29.49 | 0.9975 |
| PB-MHBs-3 | 0.014345 | 41.15 | 0.9953 |

Table S2. Cs removal performance of control MHBs (without PB) at various initial concentration of Cs.

| *C_o_* | *C_f_* | *q_e_* | *Removal efficiency* | *K_d_* |
| --- | --- | --- | --- | --- |
| 21.264 ppm | 21.088 ppm | 0.176 mg/g | 0.827% | 8.346 |
| 405.488 ppm | 404.456 ppm | 1.032 mg/g | 0.255% | 2.552 |

Table S3. Leaching of Fe after suspending 10 mg of the PB-MHBs-3 in 10 mL of different fluids for various incubation times.

|  | 1 day | 4 days | 1 week | 2 weeks |
| --- | --- | --- | --- | --- |
| Fe conc. | 0.352 ppm | 0.543 ppm | 0.678 ppm | 0.656 ppm |
| %Fe leached  from the total Fe of the PB-MHBs-3 | 0.158 % | 0.244 % | 0.304 % | 0.294 % |


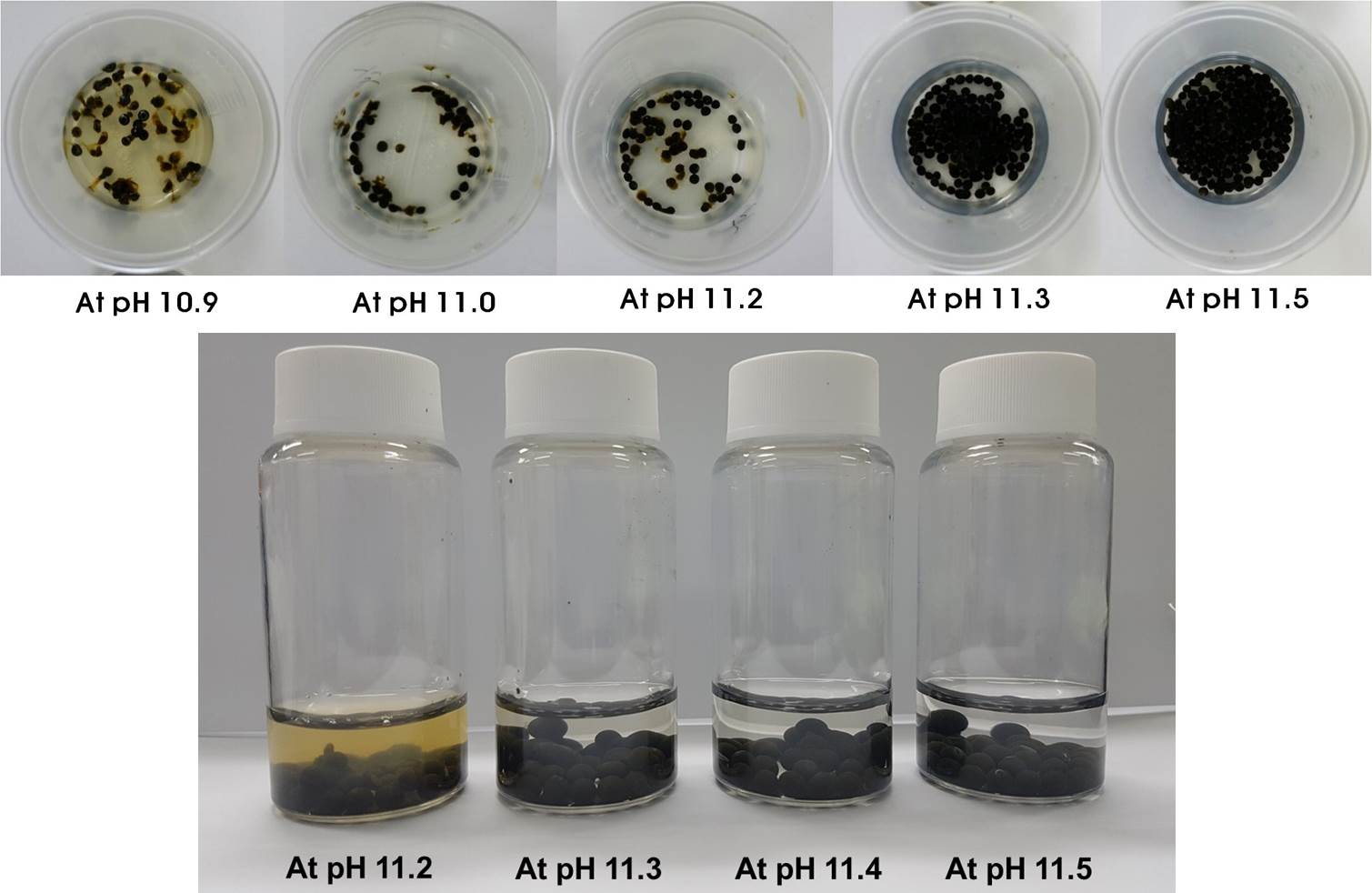


Figure S1. Photographs of PB-MHBs after reaction with solutions of NH4OH with different pH values.

*
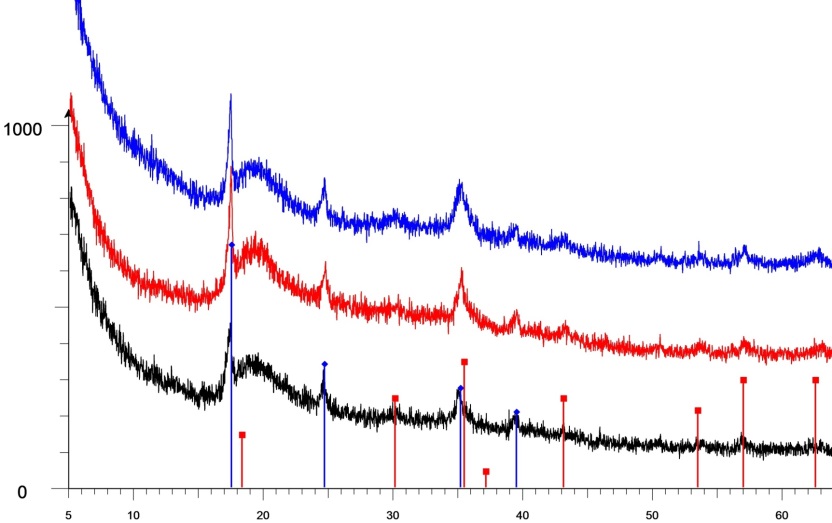
*

Figure S2. X-ray diffraction patterns obtained from PB-MHBs fabricated via reaction with NH4OH solutions with pH values of 11.3 (black), 11.4 (red), and 11.5 (blue) (red line: magnetite, blue line; PB).

*
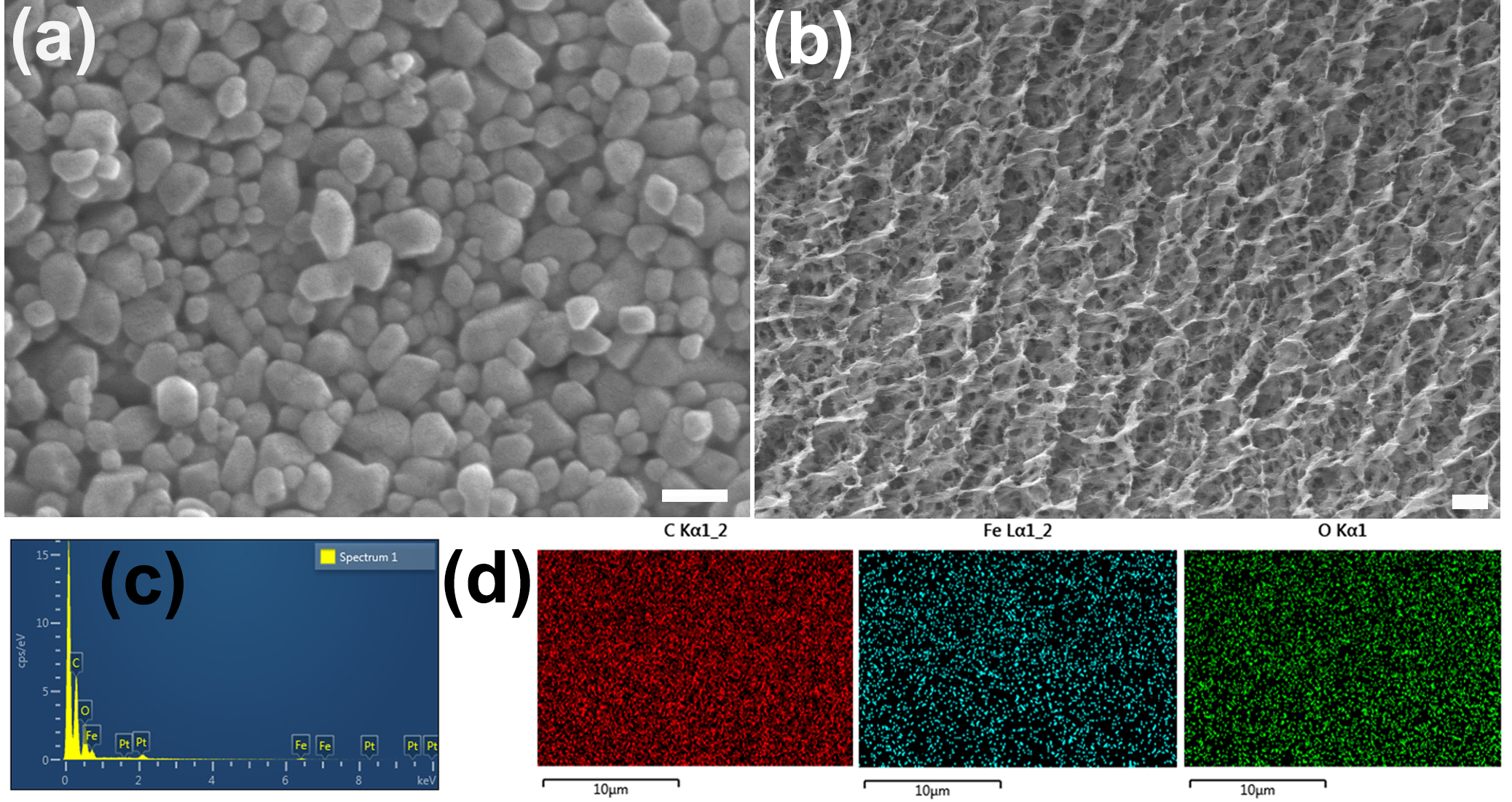
*

Figure S3. Scanning electron microscopy (SEM) images of (a) commercial PB and (b) MHBs (without PB) (scale bar : 1µm). (C) EDX data, and (d) EDX mapping of MHBs (without PB).

Figure S4. Magnetization curves obtained from MHBs and PB-MHBs with different weight ratios of PB to PVA fabricated at pH 11.3.


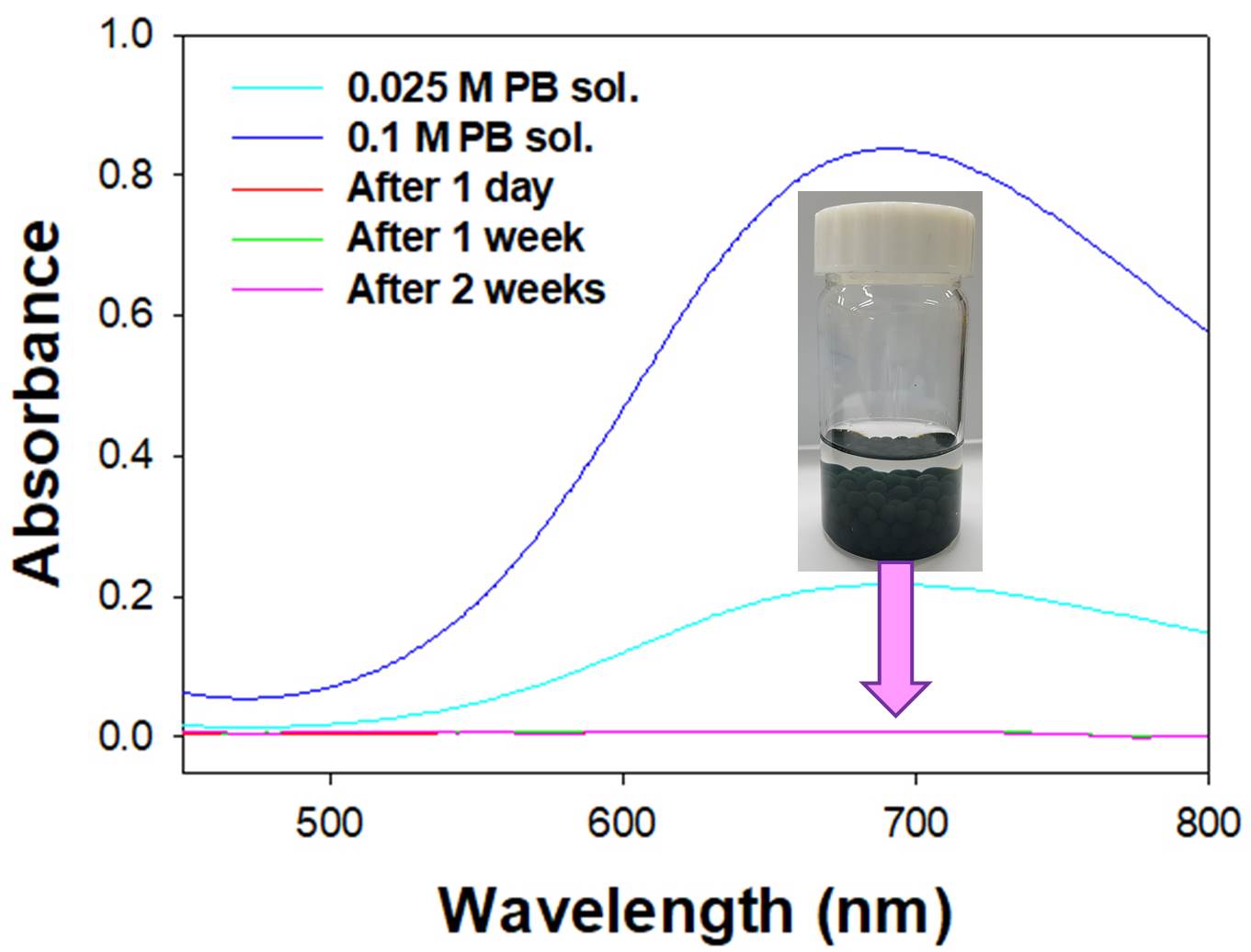


Figure S5. UV-vis spectrum of 0.025 M, 0.1 M PB solution (control), and aqueous solution after suspension of PB-MHBs-3 for various times (inset shows a photograph of PB-MHBs-3 suspended in water for 2 weeks).


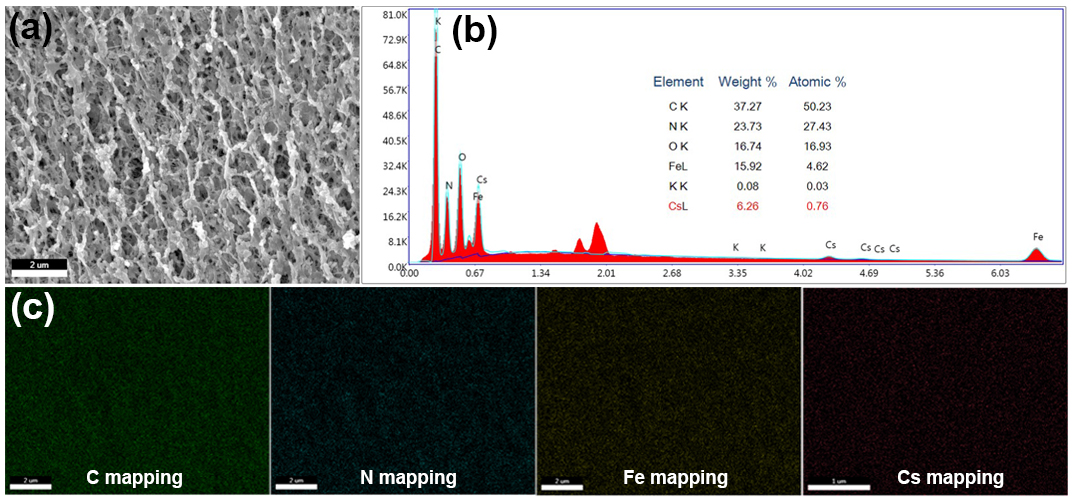


Figure S6. (a) SEM image of PB-MHBs-3 after adsorption reaction with Cs; (b) the corresponding EDX data; and (C) EDX mapping.


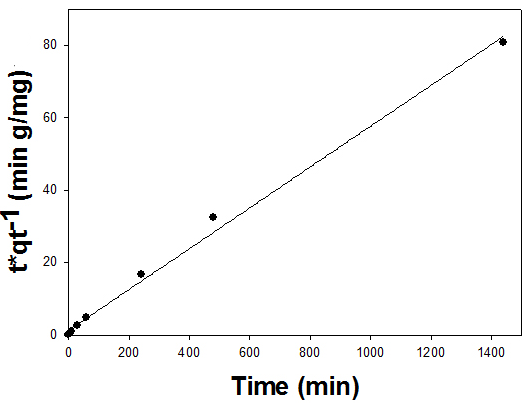


Figure S7. Pseudo-second-order model of Cs adsorption by the PB-MHBs-3.
